# Optical Coherence Tomography: Basic Concepts and Applications in Neuroscience Research

**DOI:** 10.1155/2017/3409327

**Published:** 2017-10-29

**Authors:** Mobin Ibne Mokbul

**Affiliations:** Notre Dame College, Motijheel Circular Road, Arambagh, Motijheel, Dhaka 1000, Bangladesh

## Abstract

Optical coherence tomography is a micrometer-scale imaging modality that permits label-free, cross-sectional imaging of biological tissue microstructure using tissue backscattering properties. After its invention in the 1990s, OCT is now being widely used in several branches of neuroscience as well as other fields of biomedical science. This review study reports an overview of OCT's applications in several branches or subbranches of neuroscience such as neuroimaging, neurology, neurosurgery, neuropathology, and neuroembryology. This study has briefly summarized the recent applications of OCT in neuroscience research, including a comparison, and provides a discussion of the remaining challenges and opportunities in addition to future directions. The chief aim of the review study is to draw the attention of a broad neuroscience community in order to maximize the applications of OCT in other branches of neuroscience too, and the study may also serve as a benchmark for future OCT-based neuroscience research. Despite some limitations, OCT proves to be a useful imaging tool in both basic and clinical neuroscience research.

## 1. Introduction

Advances in biomedical engineering have made several imaging modalities to be an integral part of everyday neuroscience research. Robust efforts of scientists from all over the world consequently resulted in the development of brain imaging modalities such as magnetic resonance imaging (MRI), functional MRI (fMRI), positron emission tomography (PET), electroencephalography (EEG), and near-infrared spectroscopy (NIRS) [1, 2]. Existing techniques are playing a critical role in visualizing, quantifying, and understanding brain morphology and function in both clinical and experimental studies. Now both basic and clinical neuroscience research are somehow dependent on the utility of neuroimaging methods. To make the best use of these methods' potentials and to overcome their limitations by either improving existing methods or developing new methods have become a fundamental phenomenon today.

Rodent models are crucial to the understanding of how blood flow responds to different structures of the human brain, in either a healthy or a diseased state [3]. A good number of neuroimaging methods have been developed in this regard, but they have one or more limitations of the following: they have a low spatiotemporal resolution, they are expensive, they need the use of contrast agents, they have a shallow depth, they are impractical for rodent brain imaging, they are limited in superficial two-dimensional (2D) images, and so on [3]. Consequently, there has been a growing need of an* in vivo* noninvasive label-free imaging method with a micrometer-scale spatial resolution and with a good temporal resolution along with comparatively less expensive detection system.

Optical coherence tomography (OCT) was first developed by Fujimoto's group at Massachusetts Institute of Technology (MIT) in 1991 that works based on tissue backscattering properties [4]. OCT takes the advantage of short coherence length of broadband light sources to perform micrometer-scale, cross-sectional imaging of biologic tissue sample. Within a short period of time after its invention in the 1990s, it became an important clinical imaging modality in several fields of biomedical science such as ophthalmology where imaging can be performed through transparent media of the anterior eye and the retina [5, 6]. As OCT uses optical sources at longer wavelengths, OCT can image highly light scattering soft tissue. OCT's applications now include cardiology [7, 8], gastroenterology [9, 10], urology [11, 12], dermatology [13, 14], dentistry [15], and even both basic and clinical neuroscience [3, 16–18]. The reason behind its gaining popularity within this short span of time is OCT's numerous advantages that are offered to researchers and clinicians. They are (i) quality images (OCT has demonstrated the ability to render images within a range of 1–10 *μ*m axial resolution usually and even submicrometer (0.5 *μ*m) resolution too [19]); (ii) imaging speed (OCT can give a temporal resolution up to milliseconds [20]); (iii) label-free imaging (OCT can give fine images of cerebral cortex without the need of any contrast agents [3, 21]); (iv) low cost (compared to some other imaging techniques, OCT is less expensive in most cases and even researchers from developing countries, where laboratories cannot afford to buy other expensive imaging systems, can use it); (v) additional functionality (while a basic OCT imaging method is able to render depth-resolved structural images of the target, more sophisticated OCT imaging strategies can provide additional functional information, such as blood flow (through Doppler OCT), tissue structural arrangement (through birefringence OCT), and the spatial distribution of specific contrast agents (through molecular contrast OCT) [19]).

It is readily apparent that OCT's unique capability has made it an appropriate modality for rodent brain imaging. However, today's applications of OCT in neuroscience research are not limited to rodent brain imaging. OCT is now being used in neurosurgery [18, 22–24] and even in several neurologic disorders [17, 25, 26] and in other branches/subbranches of neuroscience [27, 28]. The first intraoperative use of OCT in neurosurgery was demonstrated by Giese et al. in 2006 [23]. OCT has now gained popularity for intraoperative guidance during brain tumor resection which will be discussed later [18, 22]. Moreover, OCT can be performed as a noninvasive and no-contact technique over focal distances of several centimeters and it can be integrated into surgical microscopes which potentially allows a continuous analysis of the tissue in view by a tomographic image of the resection edge in microneurosurgery [6, 24, 29]. A potential application of OCT in experimental neuroendoscopy is also demonstrated by Böhringer et al. (2006) [30]. Again, in neurology, neuroimaging is central to the exploration for a biological foundation of psychiatric diagnosis, but so far it has not yielded clinically relevant biomarkers for mental disorders [1]. However, there has been a recent trend to establish retina as a reliable and clinically relevant biomarker and outcome measure for several neurological disorders [17, 26]. This important point enabled OCT to be a reproducible, reliable, and quick test for retina-derived biomarker development of some neurodegenerative disorders. Eye and brain come from the same embryological origin and the eye's retina has unmyelinated axons and a relatively lower concentration of glial cells [17, 26]. Moreover, retina reflects brain atrophy in neurodegenerative diseases [25, 26]. Retinal data can be used to get “meaningful difference” between images within a short neurodegenerative period [17, 26]. As a result, retinal imaging has started to shed some new light on clinically relevant biomarker development of several neurological diseases. If we take into account the fact that the retina is considered a peripheral extension of the brain and both share some common features, it becomes easy to understand why OCT has become today a widespread diagnostic tool in many neurological diseases.

Early reviews by Boppart (2003) summarized the utility of OCT in neuroimaging [16] and Baran and Wang (2016) summarized the utility of OCT angiographic methods in neuroimaging and in some specific neurological diseases such as stroke and traumatic brain injury (TBI) [3]. However, this review study is intended for a wider neuroscience community. It will provide an overview of OCT's applications in several fields of neuroscience (e.g., neuroimaging, neurology, neurosurgery, neuropathology, and neuroembryology). Examples of each of them will be described. The study focused more on OCT's applications than on image processing algorithms and other basic methodological issues. This study has briefly summarized the recent applications of OCT in neuroscience, including a comparison, and provides a discussion of the remaining challenges and opportunities as well as future directions. It is the author's hope that the comparisons provided here can also be of service to the fields outside of neuroscience. The aim of the study is to draw the attention of a wider neuroscience community in order to make the best use of OCT's potentials and to serve as a benchmark for future OCT-based neuroscience research.

## 2. Basic Principle of OCT

**Figure 1:**
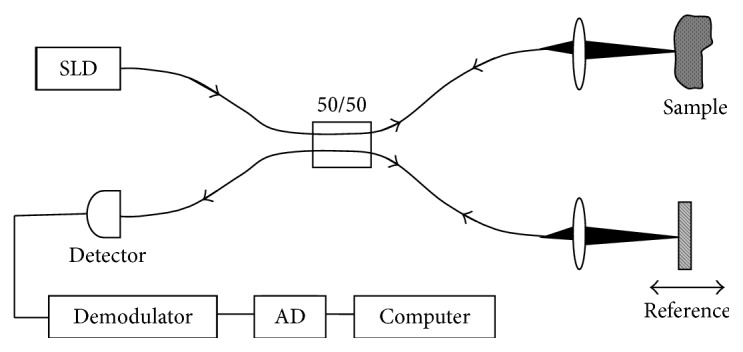
Schematic of a generic time domain OCT system (reprinted with permission from [4]).

Figure 1 shows a generic time domain OCT system. An archetypal OCT system contains a low-coherence broadband light source. The emitted light is coupled into an interferometer. Then the light is divided into two arms: reference arm and sample arm. The reference arm transmits the light toward a reference mirror. The sample arm sends the light toward the tissue of interest. The sample arm also contains an objective lens which focuses the light onto the sample tissue (e.g., brain, retina, and carotid artery). The light which is backscattered from the tissue structures is recombined with the reference light reflected from a highly reflective (>95%) moving reference mirror, producing an interference pattern that is detected by a light detector. To reconstruct the two-dimensional (2D) or three-dimensional (3D) cross-sectional objects, the beam is scanned across the sample surface. More complex systems may include a CCD camera and diffraction grating.

Since the initialization of OCT, two types of OCT implementations have been introduced: time domain OCT (TD-OCT) and Fourier domain OCT (FD-OCT) [3]. Besides, FD-OCT has two versions: swept source OCT (SS-OCT) and spectral domain OCT (SD-OCT). A typical SD-OCT scheme is very similar to that of a typical TD-OCT scheme (Figure 1). The difference is that the moving reference mirror in Figure 1 is immobilized, and the detector in Figure 1 is replaced by a low-loss spectrometer in an SD-OCT scheme. In comparison between these two versions, SD-OCT provides a significantly more detailed microstructure of brain tissues than conventional TD-OCT [32]. SD-OCT has a higher OCT scan acquisition rate, better sensitivity, enhanced signal-to-noise ratio (SNR), and superior depth penetration or improves the sensitivity of the various functional OCT methods [19]. There are some functional OCT extensions as well such as multicontrast OCT (MC-OCT) [25], polarization-sensitive OCT (PS-OCT) [33], Doppler OCT (D-OCT) [34], dynamic contrast OCT (Dyc-OCT) [35], second harmonic OCT [36], and then most recently molecular imaging true-color spectroscopic OCT (METRiCS OCT) [37]. Optical coherence microscopy (OCM) [13], optical microangiography (OMAG), and so on [3] are some other complex versions based on OCT principle.  Table 1 represents a short description of the basic principles of some of the OCT techniques used in neuroscience research so far.

## 3. OCT in Neuroimaging

Current neuroimaging methods like CT scan, MRI, and PET scan provide excellent images of the brain but those images often lack the spatial resolution and imaging speed that is required to image in cellular/neuronal level in real time [39]. On the other hand, OCT can provide high-resolution cross-sectional and volumetric images of nerve fiber bundles at real-time imaging speed [40].  Table 2 shows a short comparison of spatiotemporal resolution of OCT with some other neuroimaging techniques.

### 3.1. Neuroanatomical Imaging

**Figure 2:**
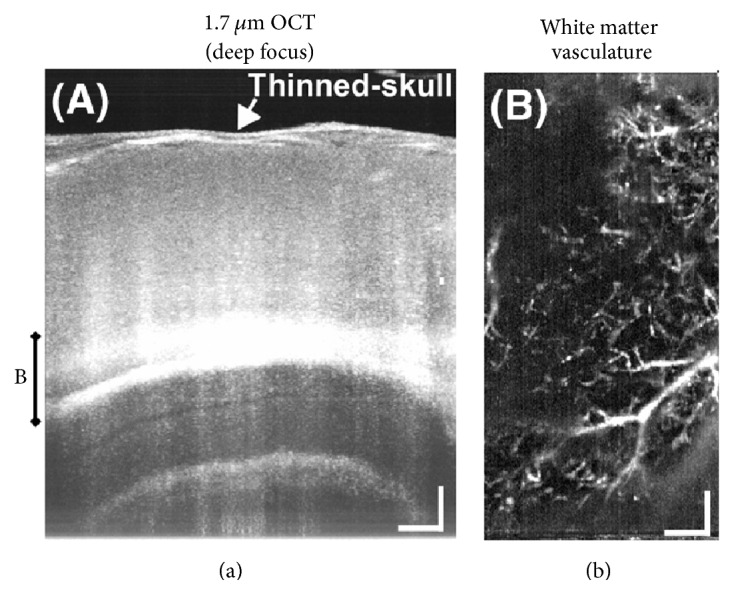
In vivo imaging of mouse brain subcortical regions noninvasively through the thinned skull by deep focusing using 1.7 *μ*m OCT. (a) A maximum intensity projection of a series of cross-sectional images shows subcortical structures, including the hippocampus proper. (b) Microvasculature in deep white matter regions is visualized using an OCT angiography method and a maximum intensity projection. Scale bars: 0.2 mm (reprinted with permission from [38]).

OCT has bridged the gap between classical macroscopic methods (e.g., MRI, ultrasounds) and shallow depth microscopic methods (e.g., confocal microscopy, two-photon microscopy) with its micrometer-scale resolution and 2-3 mm imaging depth. For instance, Watanabe et al. (2011) reported* in vivo* 3D visualization of the layered organization of a rat olfactory bulb (OB) by an SS-OCT system [41]. However, before this approach, with methods such as MRI, or confocal microscopy, OB depth structure* in vivo* had not been clearly visualized as these do not satisfy the criterion of simultaneously providing micron-scale spatial resolution and imaging up to a few millimeters in depth. Chong et al. (2015) presented an* in vivo* noninvasive OCT imaging platform in order to image deep subcortical brain regions in living mouse with a spatial resolution of 1.7 *μ*m (see Figure 2) [38].  Figure 2(a) shows mouse hippocampal image with 1.7 *μ*m resolution and Figure 2(b) shows white matter vasculature by OCT angiography performed in the same mouse brain. This imaging platform is also supposed to have applications to monitor disease progression and pathophysiology in rodent models of Alzheimer's disease and subcortical dementias, including vascular dementia.

Rat somatosensory cortex refractive index has been also measured by full-field OCT (ff-OCT) [42]. OCT has been used in label-free imaging of single myelin fibers in living rodents that required a time-consuming and invasive histological method in the past [43].

A fundamentally unsolved question in neuroscience is how the neurons are coordinated and communicated with architectural pathways and dynamic circuits to form perception, thought, emotion, and motion [44]. As a result, understanding the neural connectivity has become crucial and it is pressing a need for micrometer-scale advanced brain imaging methods. Early studies by Nakaji et al. (2008) demonstrated the utility of polarization-sensitive OCT (PS-OCT) to image micrometer-meter scale nerve fiber pathways in brain [40]. Recently multicontrast OCT (MC-OCT) and serial optical coherence scanner (SOCS) have come into spotlight for quantitative investigations of fiber orientations and connectome studies of human and nonhuman primate brains [33, 44, 45]. In addition, there is still no technology that can be used to acquire microscopic images in undistorted 3D space for mapping human brain connectivity. At present, PS-OCT has gained much attention from the investigators for high-resolution ex vivo imaging of the human brain connectome. Very recently, Boas et al. (2017) presented a possibility of developing a new imaging platform—known as automatic serial-sectioning PS-OCT (as-PSOCT) for ex vivo human brain imaging, with which it is possible to resolve human neuronal fiber projections and orientations, with 3.5 *µ*m in-plane resolution [46]. Though this novel technique requires further improvements in image acquisition rate, the method holds promise to give an improved understanding of normal human brain structure and function and of the effects of neurological disorders at cellular resolution.

However, OCT can image only up to a depth of 2-3 mm. As a result,* in vivo* noninvasive structural imaging of human brain is still not possible. It should be remembered that OCT cannot fully substitute MRI or other established techniques but can serve as a supportive tool only in experimental studies of small animal models.

### 3.2. Neurophysiological Imaging

**Figure 3:**
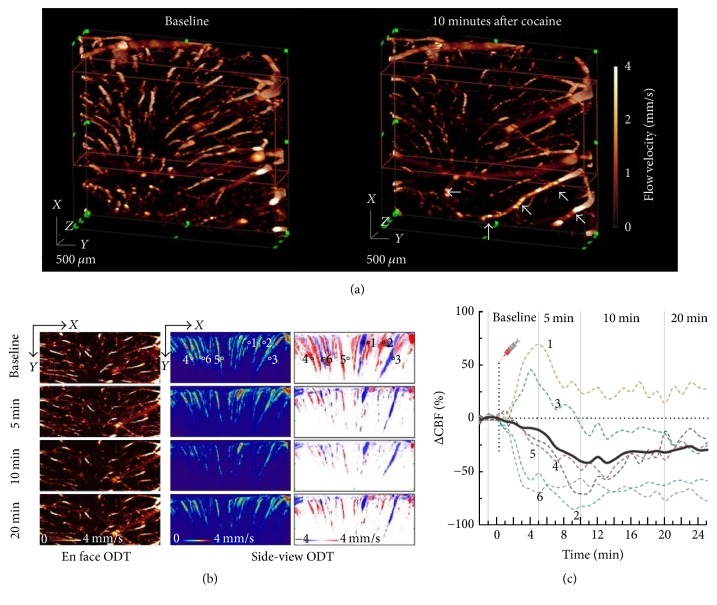
3D ODT images of CBFv in mouse somatosensory cortex due to acute cocaine exposure. (a) Dynamic changes of 3D CBF networks (FOV: 3 × 3 × 1.8 mm^3^) in mouse somatosensory cortex in response to acute cocaine (30 mg/kg,* i.p.*). (b) En face and cross-sectional projections to show the flow dynamics in different vessels (e.g., 1–6) after cocaine. (c) Time-lapse CBF change (ΔCBF%) to track the dynamics of different vascular compartments (e.g., arterioles and venules) in response to cocaine (reprinted from [47]).

In experimental settings, OCT neuroimaging has become crucial in studying cerebral blood flow (CBF), cortical hyperemia, capillary perfusion, intracellular motility, oxygen saturation, and other structural and functional changes within intrinsic contrast in living rodents [48–50]. OCT is the first accepted imaging method for* in vivo* longitudinal monitoring of CBF with high resolution in rats and mice [45]. Doppler OCT (D-OCT), which is also known as optical coherence Doppler tomography (ODT), has shown great promise in noninvasive microvascular imaging and is being widely used to investigate absolute CBF measurements in the rodent brain [34, 45]. D-OCT or ODT derived data offer great leverage in studying brain functional activation and cerebrovascular physiology [34, 51]. Moreover, D-OCT also has potentials to assist in the testing of pharmacological agents in animal models [45]. However, high-speed microcirculatory imaging in deep brain with D-OCT or ODT remained an open quest. Recently, Chen et al. (2016) have developed a 1.3 *µ*m high-speed swept source ODT (SS-ODT) system that was capable of detecting high-speed microcirculatory blood flow elucidated by acute cocaine in deep brain (see Figure 3) [47]. Figure 3 shows high-speed SS-ODT images for functional imaging of the CBF network dynamics in response to an acute cocaine challenge. Such imaging ability to differentiate CBF dynamics can be of interest for understanding complex relationship between brain function, behavior, and CBF dynamics across different cortical layers and regions as well as in different vascular trees.

Chong et al. (2015) presented a method of measuring cerebral metabolic rate of oxygen (CMRO_2_) by combined D-OCT and S-OCT [52]. Park et al. (2015) presented ODT's use in high-resolution angiography of the cerebral vasculature and quantitative CBF velocity (CBFv) in order to* in vivo* monitor neurovascular changes through cranial window due to chronic cocaine exposure [53]. This methodology can be used on animal models to explore the neurovascular functional changes induced by the brain diseases such as drug addiction. Optical coherence microangiography (OMAG) has been used to image label-free* in vivo* imaging of capillary level microcirculation in the meninges in mice with the cranium left intact [54]. Besides, OCT is also being widely used in studying brain functional activation by some research groups [55, 56].

Other neurophysiological parameters such as oxygen saturation, hemoglobin concentration, cerebral oxygen delivery, energy metabolism, and red blood cell (RBC) flux are studied using OCT [20, 57, 58]. Another important neurophysiological parameter, capillary transit time distribution, was challenging to quantify comprehensively and efficiently at the microscopic level in the past [35]. Recently Merkle and Srinivasan (2016) have used dynamic OCT (dyc-OCT) to investigate capillary transit time distribution in microvasculature across the entire depth of the mouse somatosensory cortex [35]. The findings may aid in explaining the time kinetics of the blood-oxygen-level-dependent functional magnetic resonance imaging (BOLD fMRI) response.

Nevertheless, there are some limitations as well in OCT neurophysiological imaging. OCT angiography cannot make accurate measurements for those vessels smaller than 20 *μ*m [3]. This problem may be mitigated by a cost-effective higher-resolution system with sufficient depth of focus to cover the entire cortical layer. But this is only the current limit, and different OCT setups are under development which can reach a resolution up to 1 *μ*m. Again, in experimental settings, thinned skull technique for OCT studies creates some subdural hemorrhage due to vibration of drilling and interferes from time to time [54]. The remaining thinned skull lacks vasculature, it starts to become opaque within hours, and thinning needs to be repeated if more imaging sessions are followed days after. To remove this limitation, cranial window and catheter probes can be used. In addition, D-OCT and other OCT angiographic methods (e.g., OMAG) can measure only axial velocity and typically fail to detect the RBC velocities [3].

### 3.3. Neural Activity Imaging

Neural activity in a neural network is mostly characterized by action potential (AP) propagation and generation through nerves [39]. APs are generated when the nerves are excited by a stimulus either as an external input or as a means of internal communication between nerves. Neural probes with high spatial resolution are needed for both neural recording and stimulating specific functional areas of the brain with precision. With multiple advantages, existing neural recording methods have some limitations which are shortly mentioned in Table 3 [59]. Therefore, there has been a budding need for high spatiotemporal imaging technique for the functional imaging of brain activity especially from individual neurons with miniaturized less expensive detection systems.

The utility of optical instrumentation to study neural activity dates to the late 1940s [60]. However, Cohen (1973) first pioneered the investigation of optically detecting birefringence, fluorescence, light scattering, and structural changes during AP propagation [61]. These intrinsic optical signals can be observed with optical methods such as Doppler flowmetry (LDF), near-infrared (NIR) spectrometer, functional optical coherence tomography (fOCT), and surface plasmon resonance (SPR) [59]. However, intrinsic optical signals are very small most often, which has entailed the use of molecular probes [62]. Consequently, fast voltage-sensitive dyes are being widely used to enhance the signal-to-noise ratio (SNR). For this reason, though OCT can render label-free imaging of neural activity [39, 60, 62], voltage-sensitive dyes are also used in most cases.

**Figure 4:**
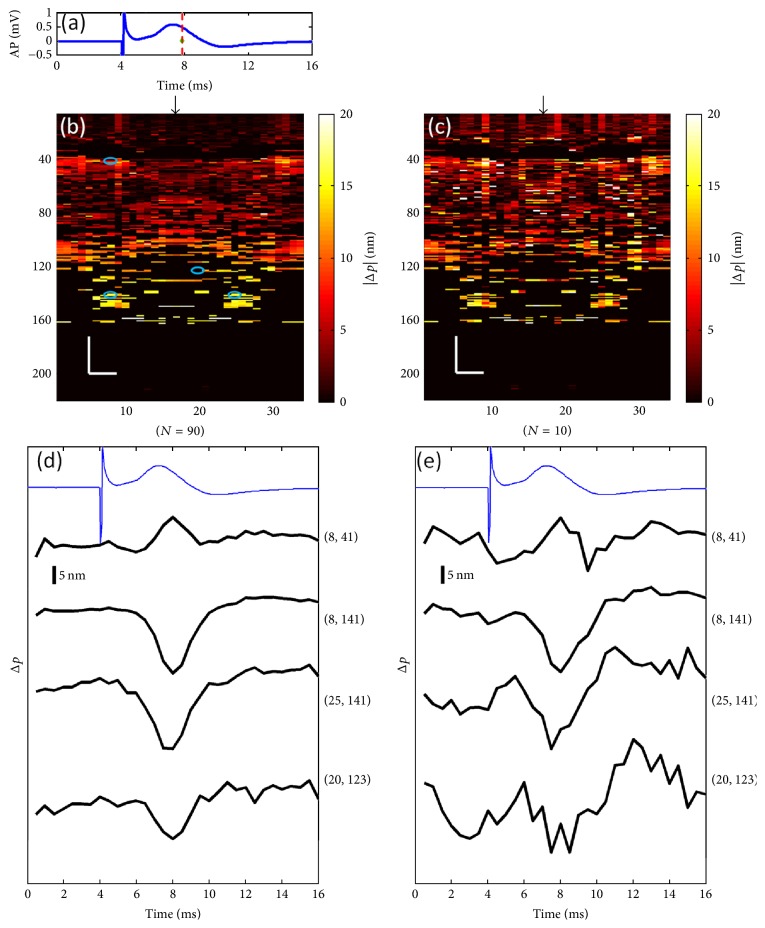
Details of the Δ*p* response in a stained squid nerve. (a) Action potential; (b) |Δ*p*| response at ∼8 ms is given by averaging 90 trials (*N* = 90) and (c) 10 trials (*N* = 10). In (b) and (c), arrow indicates the turning point of the galvanometer, and pixel numbers are given on the *x*-axis and *y*-axis. Scale bars: horizontal, 10 *μ*m; vertical, 100 *μ*m. Signal traces in time (d, e) are given for selected pixels (lateral index, depth index), which are marked by the blue circles in (b), with averaging 90 and 10 trials. Action potential traces in blue are for guidance (reprinted with permission from [62]).

**Figure 5:**
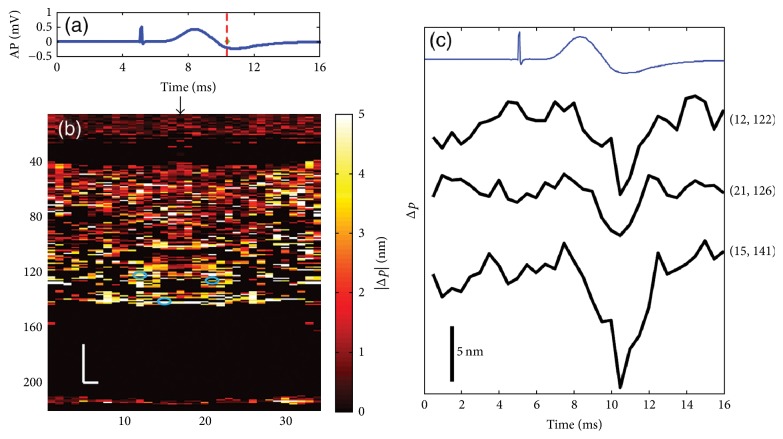
Details of the Δ*p* response in an unstained squid nerve. (a) Action potential recording; (b) |Δ*p*| image at ∼10.5 ms and (c) Δ*p* traces of selected pixels from an unstained nerve with an averaging of 90 trials. Arrow in (b) indicates the turning point of the galvanometer mirror; scale bars: horizontal, 10 *μ*m; vertical, 100 *μ*m. Pixel coordinates in (c) are in the form of (lateral index, depth index), and the locations are marked by blue circles in (b). The blue trace is the action potential recording for the registering time (reprinted with permission from [62]).

Akkin et al. (2010) have reported an SD-OCT intensity measurement on a squid giant axon with and without being stained with voltage-sensitive dye to localize neural activity in depth [60]. Yeh et al. (2015) have proposed a functional OCT scanner to detect cross-sectional neural activity in unmyelinated nerves dissected from squid (see Figures 4 and 5) [62]. They conducted the study on both stained and unstained nerves and detected transient phase changes from backscattered light during AP propagation. Figures 4 and 5 show action-potential-related optical path length changes (Δ*p* response) in stained and unstained nerves. Their imaging method increased the number of recording sites to yield neural activity in optical cross sections at high resolution, which may support neurophysiological studies in the future.

Watanabe et al. (2011) used SS-OCT to guide electrode penetration in neural tissue for* in vivo* neural recording [63]. This also suggested that the SS-OCT penetration system may aid future* in vivo* microinjection studies in neural tissue.

OCT neural recording may have a significant impact on functional neuroimaging in near future [39, 60]. It can be foreseen that OCT would enable the investigators to compare the local structural and functional changes with a high spatiotemporal resolution during AP propagation, and be used as an alternative or supportive tool to electrophysiology. It has the potential to produce critical data, which could increase the understanding of functioning nerve and aid future diagnostic applications [60]. However, OCT is unable to detect functional changes or neural activity in the freely moving animal because of its bulky system. Further development is required in miniaturization of OCT system along with portability for neural recording and improvement of sensitivity.

## 4. OCT in Neurology

OCT has been used in answering several clinical questions of neurology. Apart from experimental neuroimaging studies mentioned above, recent innovations in establishing retina as a biomarker to several neurologic disorders have enabled OCT to contribute bravely to clinical neurology too. Early studies suggested that several neurologic conditions have pathologic changes in the retinal nerve fiber layer (RNFL) of the eye, creating a potential biomarker for neurodegeneration [17]. Utilizing data that can be gathered from examinations of the eye has allowed novel insights into the neurologic disease to be garnered and modeling systems to be developed. Thus, OCT has the potential to become a noninvasive, reproducible test for axonal degeneration and could become an invaluable tool for measuring the efficacy and safety of potential neuroprotective agents. Future neurologic clinical trials may incorporate OCT data in the outcome measures for drug validation. Apart from retinal imaging of neurologic disorders, OCT is also being used in other subbranches of neurology and even in neurosurgery which is discussed below.

### 4.1. Neurological Disorders

In clinical settings, the major users of OCT technology over the last 20 years have been mostly ophthalmologists, but in these days, it is also being used by neurologists on patients with neurologic disorders [17]. An early review by Greenberg and Frohman (2010) has excellently summarized why and how OCT is contributing to clinical neurology [17]. A number of neurologic diseases follow a degenerative course, and neurological diagnosis is dependent on MRI technology in this regard, but its reproducibility as an outcome measure has been limited (so called clinic-radiological paradox) [17, 64]. Moreover, in some diseases, MRI is practically useless for structural documentation and measuring disease progression [17]. For this reason, in clinical trials, outcome measures have mostly depended on clinical assessment scales that usually need some years to measure “meaningful differences” between samples [17]. These caveats necessitated the development of a reproducible and reliable diagnostic modality to gather “statistically significant” changes in either retina or brain over a short degenerative period in clinical assessment. Though brain and retina come from the same embryologic origin, retina provides a unique window into the nervous system because of having unmyelinated axons and a low concentration of glial cells. That is why retina is called “a relative vacuum” while studying neurons and axons and it can serve as a valuable surrogate marker of neurodegeneration, neuroprotection, and neurorestoration [17].

Table 4 represents the current status of neurological diseases studied (clinically or nonclinically) with OCT. Those studies (in brain or retina) include understanding pathologic changes, clinically relevant diagnostic biomarker development, or drug trials.

**Figure 6:**
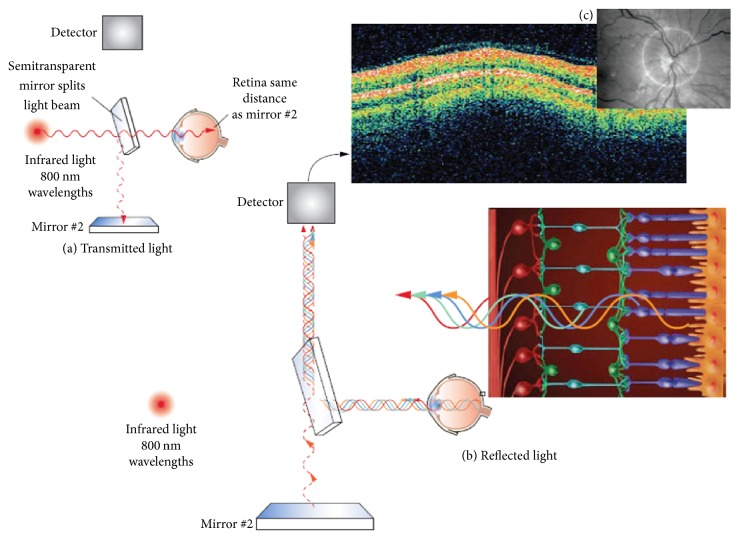
High-resolution images of the internal retinal structure taken with optical coherence tomography (OCT). (a) Low-coherence infrared light is transmitted into the eye through use of an interferometer. (b) The infrared light is transmitted through the pupil and then penetrates through the nine transparent layers of the retina. (c) A fundus image from the optical coherence tomography (OCT) device showing the optic disc appropriately centered and surrounded by the target image circumference marker for analysis of the retinal nerve fiber layer (reprinted with permission from [64]).

Figure 6 demonstrates an illustrative summary of the imaging operation involved in OCT retinal study of neurologic diseases. In usual OCT retinal imaging setup, high-resolution cross-sectional or three-dimensional images of the internal retinal structure are generated by an optical beam being scanned across the retina or by a circular scan around the optic nerve [64]. OCT captures cross-sectional images of the retina from a series of lateral adjacent depth scans. OCT, therefore, allows direct visualization and measurement of RNFL thickness and macular volume with micron-scale resolution. The first neurologic disease studied with OCT is Multiple Sclerosis (MS). In MS, retinal OCT imaging has already taken a role of similar importance to the retina as the radiological imaging for the brain [83]. Changes in the RNFL and macula reflect mechanisms of inflammation, demyelination (retrobulbar), axonal degeneration, and neuronal degeneration in MS that can be observed in ophthalmic imaging [64, 65]. As a result, OCT has gained a high clinical utility in predicting MS disease course and determining treatment response. Likewise, other neurological diseases mentioned in Table 4 which also show significant changes in the eye can be evaluated in the same way. Thus, OCT opened a new avenue of research in clinically relevant biomarker development and potential surrogate biomarkers development of early-stage neurodegenerative disorders and for neuroprotective drug trials.

However, there are some limitations. OCT cannot robustly differentiate between AD and non-AD dementias (only 40%) [90]. Establishing OCT-derived retinal data as a regular diagnostic gold standard of neurodegenerative diseases in hospitals and clinics is still a challenge. Most of the OCT studies in this regard targeted a single parameter [91]. More extensive and multiparametric longitudinal cohort studies are required in larger patient groups. More studies should correlate OCT-derived data with MRI and other clinical tests (e.g., physical evaluations, cognitive tests). Future studies should, therefore, include prospective and long-term studies including patients with different subtypes of each neurologic disorder, confirmation of the relationship between MRI markers of disease activity, including RNFL, and clinical outcomes, and the assessment of RNFL thickness in response to treatment. Technical improvements on OCT system are required such as real-time eye tracking system, automatic optic disc centering, coregistration capability, and eye movement correction analysis.

**Figure 7:**
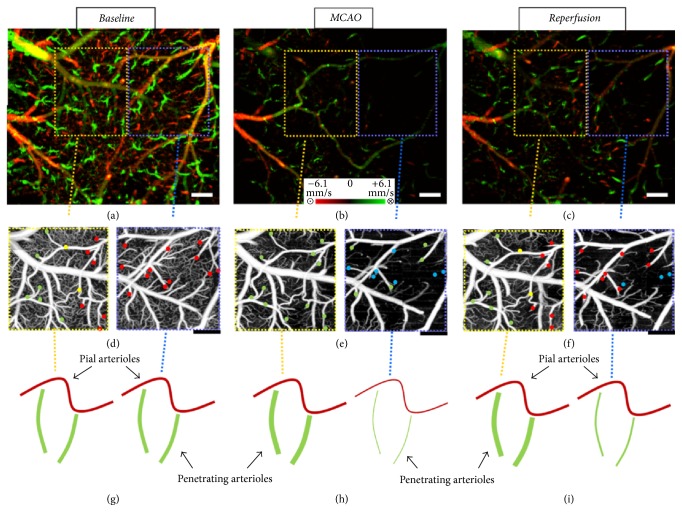
Comparison between regions where AAA is relatively stronger or weaker. (a–c) DOMAG results for (a) basal, (b) during-MCAO, and (c) after-reperfusion conditions, respectively. Strong AAA area is marked with a yellow dashed box and weak AAA area with a blue dashed box. (d–f) OMAG comparison between strong and weak AAA ROIs for (d) basal, (e) during-MCAO, and (f) after-reperfusion conditions, respectively. (g–i) Red, green, and yellow dots correspond to MCA, ACA, and AAA T-junction sourced arterioles, respectively. Blue dots correspond to the diving arterioles that are at nondetectable level compared to basal condition. Cartoon representations of the lumen diameters of pial and penetrating arterioles for (g) basal, (h) during-MCAO, and (i) after-reperfusion conditions, respectively. Scale bar is 0.3 mm (reprinted with permission from [77]).

Another field of research with OCT is rodent model of experimental ischemic or hemorrhagic stroke and traumatic brain injury (TBI) [78, 79, 83]. Stroke leads to a series of functional and structural changes that may result in an infarct or a peri-infarct brain tissue (also called penumbra) [3]. But penumbra is potentially recoverable if it can be identified early and treated appropriately. Recently, with OCT angiography, Baran et al. (2015) have discovered the functional role of arterio-arteriolar anastomosis (AAA) in the active regulation of the pial and penetrating arterioles during the stroke [77]. AAA provided blood flow to actively dilate the penetrating arterioles in order to rescue the tissue in the penumbra region (see Figure 7). Figure 7 shows AAA's role in the dilation of the pial and penetrating arterioles to middle cerebral artery occlusion (MCAO) with both Doppler OMAG (DOMAG) and OMAG results. They found that abundance of anastomosis may prevent or delay permanent neural damage after stroke and thus gives guidance to clinicians to reduce stroke damage with a biologically initiated system. It has been considered as one of the major discoveries of stroke recovery.

Besides, OCT angiography has been used to study subarachnoid hemorrhage in TBI and neovascularization and even in assessing the therapeutic effect of soluble epoxide hydrolase (sEH) gene deletion in revascularization after TBI [3, 83, 84]. Moreover, the ability to noninvasively map the variations of CBF over a long period of time using OCT angiography would contribute to the understanding of the complex mechanisms related to TBI recovery [3]. Some research groups are utilizing OCT to detect cortical optical changes during seizure activity [92, 93]. As it is a relatively newer field of research, future studies should compare the temporal performance of seizure detection with OCT with that of gold standard EEG.

### 4.2. Neurosurgery

Intraoperative neurosurgery guidance plays a key role in conducting a neurosurgery. Intraoperative neurosurgery guidance has seen significant advancements since the introduction of microelectrode recording (MER) and intraoperative MRI (iMRI) [18]. MER is an accurate tool for locating brain nuclei by observing the distinctive neuron firing patterns, but it also increases the risk of disastrous intracranial hemorrhage, requires long operation hours, and is not capable of targeting white matter tracks [18]. iMRI, in contrast, is capable of locating different tissues (lesions, nuclei, and white matter tracks) and avoiding hemorrhage. However, iMRI imaging suffers from low spatial resolution (1 mm), slow imaging speed (tens of minutes), and high cost. On the other hand, OCT is a promising real-time guidance tool, because it is capable of imaging brain nuclei, lesion, fiber tracks, and brain blood vessels simultaneously in real time and can detect important tissue landmarks for neurosurgery guidance [18]. OCT has been successfully used in intraoperative guidance for brain tumor resection surgery, vascular neurosurgery, and spinal cord surgery and even in other neurosurgical interventions such as deep brain stimulation (DBS) therapy [18, 64, 88–90].

The first intraoperative use of OCT in neurosurgery was demonstrated by Giese et al. in 2006 [23]. They firstly demonstrated OCT's application to the intraoperative detection of residual tumor during resection of human gliomas. Later on, OCT has also been used in intraoperative guidance during glioblastoma and other types of glial tumors resection [5, 86]. Assayag et al. (2013) performed an intraoperative clinical diagnostic assessment of meningioma, choroidal plexus papilloma, glioma, and hemangiopericytoma with FF-OCT [85]. FF-OCT's 1 *μ*m resolution and wide field down to cell level views allowed clinical identification of features of nontumorous and tumorous tissue such as myelin fibers, neurons, microcalcification, tumor cells, microcysts, and blood vessels.

**Figure 8:**
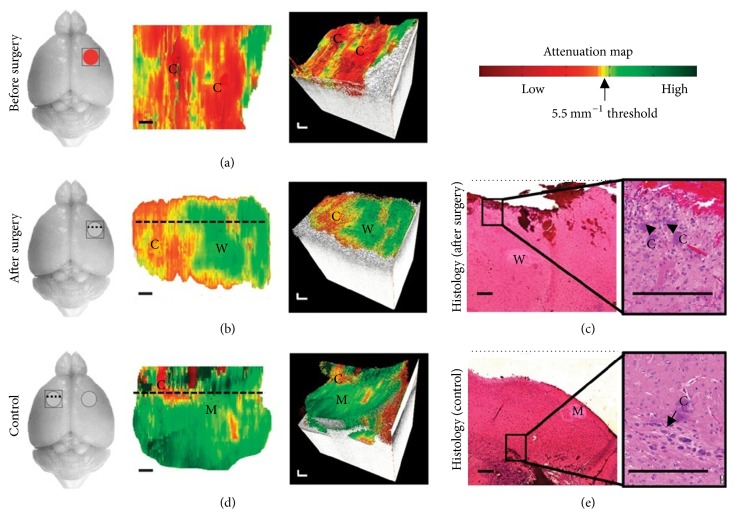
In vivo brain cancer imaging in a mouse with patient-derived high-grade brain cancer (GBM272). (a and b) Brain tissues were imaged* in vivo* in mice (*n* = 5) undergoing brain cancer resection. After imaging, the mice were sacrificed and their brains were processed for histology. Here, we show the representative results of a mouse brain at the cancer site before surgery (a) and at the resection cavity after surgery (b). (c) Corresponding histology for the resection cavity after surgery was also shown. (d and e) With the same mouse, control images were imaged at a seemingly healthy area on the contralateral, left side of the brain (d), with its corresponding histology (e). The red circle indicates cancer, gray circle indicates resection cavity, and square was the OCT FOV. 2D optical property maps were displayed using an attenuation threshold of 5.5 mm^−1^. C, cancer; W, noncancer white matter; M, noncancer meninges. Aliasing artifacts at the image boundaries, which were produced when dorsal structures from outside the OCT depth were folded back into the image, were cropped out of image. 3D volumetric reconstructions were overlaid with optical property maps on the top surface. Optical attenuation properties were averaged for each subvolume of 0.326 mm × 0.008 mm × 1.8 mm within the tissue block, with a step size of 0.033 mm in the* x* direction. Each histological image (c and e) represented a cross-sectional view of the tissue block: the image corresponds to a single perpendicular slice through the attenuation map, along the dotted lines in (b) and (d), respectively. Residual cancer cells were marked with black arrows and correspond to yellow/red regions on the attenuation maps (at the level of the dotted line). Scale bars, 0.2 mm (reprinted with permission from [22]).

In addition, a major concern in brain tumor resection is making minimal damage to healthy tissue and maximum resection of cancerous tissue and OCT attenuation map can play a crucial role in this regard. The OCT signal attenuation of brain tissue and differences between healthy and tumorous brain tissue was firstly explained by Böhringer et al. in 2009 [5]. They demonstrated that OCT analysis of the tissue microstructure and light attenuation characteristics can discriminate normal brain, areas of tumor infiltrated brain, solid tumor, and necrosis. Later on, Kut et al. (2015) demonstrated the translational and practical potential of OCT in precisely differentiating cancer from noncancer tissue with continuous guidance in rat brain neurosurgery using OCT attenuation maps (see Figure 8) [22]. Figure 8 shows the OCT attenuation maps which aided the investigators in identifying regions of cancer versus noncancer (white matter) before and after surgery, even for mice that displayed more infiltrative brain cancer characteristics with the patient-derived GBM272 cell line. They found lower attenuation values for cancer tissues in both cancer core and the infiltrated zone when compared with surrounding noncancer white matter. For high grade, the average optical attenuation value of noncancer (6.2 mm^−1^) was significantly higher than that of infiltrated zone (3.5 mm^−1^) and cancer core (3.9 mm^−1^); for low-grade, the average optical attenuation value of noncancer (6.2 mm^−1^) was significantly higher than that of infiltrated zone (2.7 mm^−1^) but not significantly higher than that of cancer core (4.0 mm^−1^). After imaging, mice brains were resected and the corresponding histological slides were reviewed for validation of the OCT results. These histological slides were sectioned in the same orientation as OCT cross-sectional images (i.e., perpendicular to the tissue surface or perpendicular to the OCT attenuation map and along the dotted lines in Figures 8(b) and 8(c)). In the postsurgery and control images, residual amounts of cancer (about 5 to 10% of the imaged area) were visible in the histological images. After performing the surgery, they found OCT resection of infiltrative brain cancers led to dramatically improved outcomes when compared with current clinical standards.

In neurosurgery, visualization with high resolution of the surgical field is important because the neurosurgeon has to examine fine structures. Therefore, the surgical microscope is a key tool in this regard, and robotization in combination with advanced control concepts could directly impact and benefit the clinical workflow [95]. OCT intraoperative approaches with integrated surgical microscopes have already gathered much interest by several investigators [24, 29, 95, 96]. Finke et al. (2012) described the combination of OCT and a robotic driven surgical microscope to enable the complete OCT scanning of a larger area in neurosurgery [96]. Moreover, in brain tumor resection, combination of OCT with a surgical microscope allows a continuous analysis of the resection plain, providing optical tomography, thereby adding a third dimension to the microscopic view and information on the light attenuation characteristics of the tissue [5]. Another approach was integrating automated neuronavigation strategies with OCT in neurosurgical settings [5, 23]. Sun et al. (2012) investigated the use of handheld OCT (HH-OCT) probe for high-resolution depth-resolved neurosurgical imaging of pituitary tumors, cerebral aneurysms, and transsphenoidal neuroendoscopic treatment of pituitary tumors [97]. Among some other most noteworthy approaches of OCT neurosurgery, combining OCT with MRI has become one of the most powerful tools for neurosurgery [18, 98]. Needless to say, prospective clinical implementation of OCT in neurosurgery may yield more precise neurooncological resections. Besides, a potential application of OCT in experimental neuroendoscopy is also demonstrated by Böhringer et al. (2006) [30]. Future studies should lay more focus on combining OCT with surgical microscopes and/or with fully motorized robotic strategy which may synergize future neurosurgical applications. Combination of OCT with endoscopes, OCT with MRI, and OCT with neurosurgical handheld probes also holds promise.

In vascular neurosurgery, OCT has already proved its superiority over intravascular ultrasound (IVUS) and other angiographic methods because of its imaging resolution [99–101]. On account of poor resolution, IVUS and other methods seemed difficult to guide carotid artery stenting, discriminating intraluminal thrombus from the tissue component, discriminating vulnerable plaques, and guiding some other neurovascular interventions [7, 99, 100]. For instance, atherosclerotic plaques in major cerebral vessels (e.g., carotid artery) are usually disrupted in acute stroke [100]. But precise imaging of vessel wall is difficult using conventional MRI, angiography, or IVUS. On the other hand, OCT can make 4D ultrahigh resolution reconstruction of vessel lumen in real time that could also influence revascularization and clipping strategies [100, 102]. Yoshimura et al. (2013) used OCT in the assessment of intraluminal thrombus before carotid artery stenting (CAS) prior to neurosurgery [99]. Hoffmann et al. (2015) utilized intravascular OCT for the evaluation of stent position, its shape, and its correct expansion of neurovascular implants (e.g., braided flow diverter, laser cut stents) in postoperative neurosurgery [102]. Moreover, OCT can also be used in carotid endarterectomies [103]. However, due to OCT's poor imaging depth, OCT cannot fully substitute IVUS or other methods. OCT can only be a complementary tool to IVUS and other established methods.

In addition, OCT has also been applied in intraoperative guidance during spinal cord interventions [104, 105]. Recently Giardini et al. (2016) presented an OCT-image guided spinal cord surgery [104]. They found that OCT probing does not interfere with the neural tissue function during spinal cord surgery. Thus OCT shows the potential to become a viable referencing technology for safer and faster minimally invasive spinal surgical guidance with intrinsic neurodynamic safety. However, it is readily apparent that a relatively fewer number of studies have explored OCT's potential in spinal cord surgery. Future studies should, therefore, compare existing gold standards with OCT in this regard.

### 4.3. Neuroembryology

The brain of a developing embryo is highly susceptible to changes caused by a number of factors, both genetic and environmental [28]. A high-resolution noninvasive imaging technique would play a vital role in understanding these changes and the causes and outcomes of various brain developmental disorders and analyze the effects of teratogens on prenatal development. Existing techniques such as histological sectioning, micro-MRI, microcomputed tomography (micro-CT), and ultrasound biomicroscopy (UBM) imaging provide fine 3D live imaging in longitudinal studies. But the lack of imaging resolution, contrast, depth penetration, or extended acquisition time limits the ability of these modalities to perform a careful quantitative analysis [94]. Thus, in experimental studies, OCT is gaining popularity for in utero embryonic brain imaging as well as contributing to developmental biology [28].

**Figure 9:**
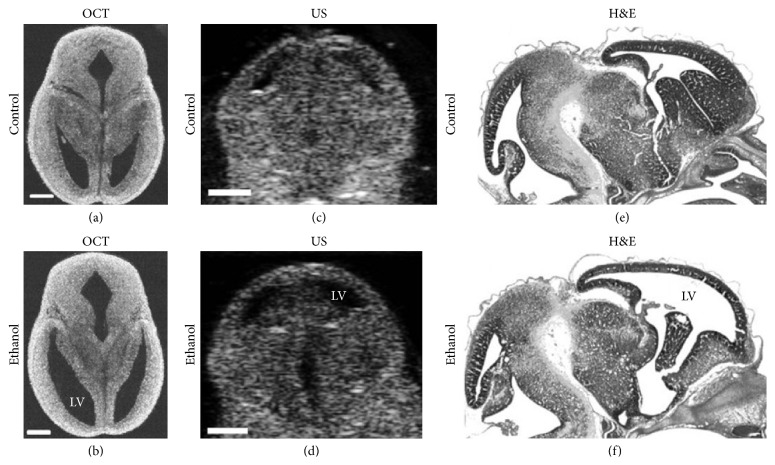
Effect of ethanol on mouse fetal brain development at GD14.5. Left panel represents OCT images (a) and (b) in horizontal section, middle panel shows US images (c) and (d) in the coronal plane, and right panel illustrates H&E images (e) and (f) in sagittal section of fetal brains from both control and ethanol treated pregnant dams. Ethanol induced increase in ventricular dilation can be observed with all the imaging techniques used in the study and in all three planes of orientation. LV, lateral ventricles; US, ultrasound; OCT, optical coherence tomography; H&E, hematoxylin-eosin staining. Scale bar, 500 *μ*m (reprinted with permission from [94]).

Earlier neuroembryological studies on* Xenopus laevis* using OCT by Boppart et al. (1996) have proved OCT's capability to provide label-free, high-resolution images of developing embryos noninvasively [106, 107]. Moreover, early cellular OCT imaging of developing embryos revealed neural crest cell's (melanocytes) motility in* Xenopus* tadpole [16]. Recent studies are focusing on in utero brain imaging on developing mice embryos [94, 108]. Sudheendran et al. (2013) investigated the effects of alcohol exposure on fetal brain development due to maternal consumption of alcohol with both OCT and ultrasonography (US) (see Figure 9) [94]. Figure 9 apparently demonstrates that the ventricles of the embryos exposed to ethanol are dilated in comparison to the controls and OCT is more accurate than ex vivo US imaging. Therefore, OCT can be a useful and sensitive imaging modality for preclinical studies, to assess the effects of drugs of abuse and other environmental teratogens. However, further more studies are required to identify the range of quantitative OCT-derived measurements that can serve as useful biomarkers for fetal teratogenesis.

Even though OCT has superior resolution and contrast, unlike US imaging, it has limited imaging depth due to light attenuation in tissues. In addition, the lack of penetration depth has limited OCT's capability in imaging depth-resolved images of deeper structures, particularly in later stage embryos. Nevertheless, Larin's research group at the University of Houston first demonstrated the possibility of imaging the developing brain structure in utero using an SS-OCT system while developing a protocol for high-resolution in utero imaging of live mouse embryos in 2011 [28, 109]. The group performed in utero brain imaging with OCT and found that the cerebral cortex and ventricles were clearly visualized until E16.5. After this stage, only the cortex was still visible. However, it is clear that a relatively fewer neuroembryological imaging studies are performed using OCT. Future studies should properly explore OCT's potential in in utero brain imaging of developing mouse embryos.

### 4.4. Neuropathology

Brain biopsy infers high risks for patients in most cases. Moreover, histological examination destroys the specimen. Conversely, OCT* in vivo* noninvasive optical biopsy enables real-time imaging with high resolution similar to a standard histological examination without the need for tissue removal [27]. Leahy et al. (2013) demonstrated that OCT brain tissue characterization (*in vivo*/ex vivo) have several advantages over conventional 2D sectioning, mounting, staining, imaging, and stereological procedures [36]. Apart from these capabilities, OCT can also guide excisional biopsies [27]. OCT is a promising imaging tool for rapid identification of suspect regions and to guide excisional biopsy while reducing the likelihood of false negatives due to sampling errors.

**Figure 10:**
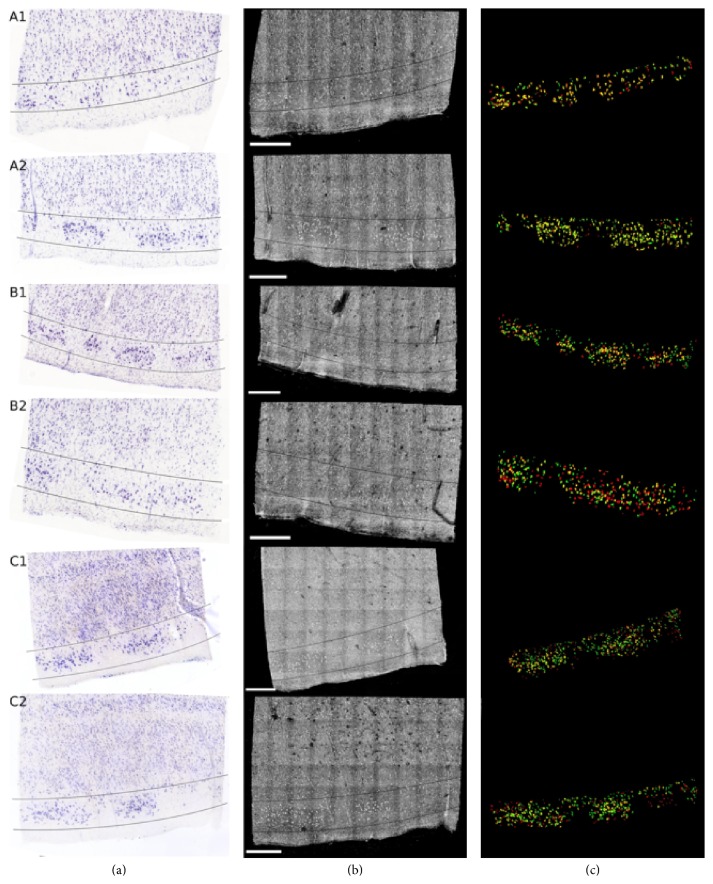
Colocalization of the neurons in layer II of EC for six different tissue samples: registered Nissl stain (a), OCT image (b), and the overlay of the segmented neurons (c): green for Nissl, red for OCT, and yellow for the overlap. Scale bar: 500 *μ*m (reprinted with permission from [110]).

Magnain et al. (2014) demonstrated that SD-OCT's capability reliably generates 3D reconstructions of multiple cubic centimeters of cortex without any extrinsic contrast agents, and it can be used to accurately and semiautomatically perform standard histological analyses [21]. They demonstrated that OCT images exhibit sufficient contrast in the cerebral cortex to reliably differentiate the cortical layers compared to gold standard Nissl staining. Later on, Magnain et al. (2015) showed that OCT can discriminate healthy neurons in ex vivo fixed human entorhinal cortex in depth (see Figure 10) [110]. They validated the technique by histological Nissl staining.

Figure 10 shows the registered Nissl stained slices (a), OCT images (b), and overlap of the segmented neurons found in layer II, delineated on the Nissl and OCT images by the lines (c) for the six tissue samples studied: green for Nissl, red for OCT, and yellow for the overlap. Thus, OCT paved the way to undistorted, high resolution, 3D visualization of the cytoarchitecture in the human cortex at the level of single neurons. Therefore, it is an important step to label-free micron-scale imaging of myelo- and cytoarchitectural properties of the entire human brain.

## 5. Limitations and Challenges

**Figure 11:**
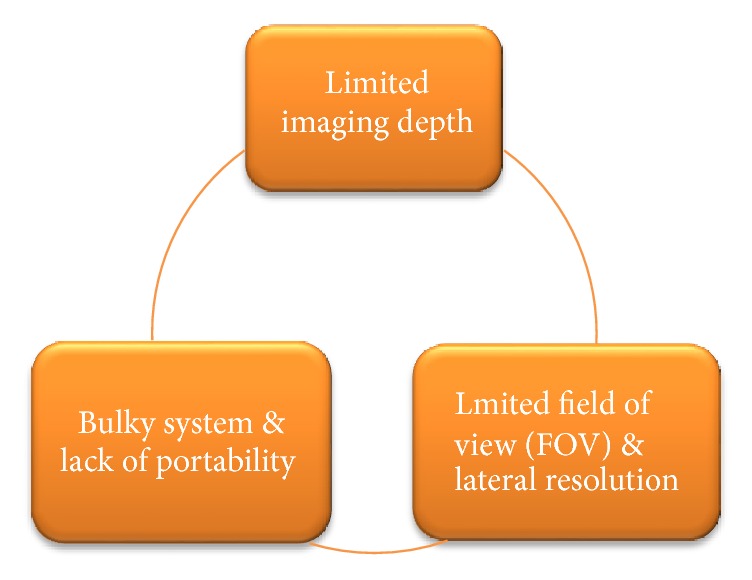
Major limitations of OCT technology.

Although OCT technology has shown its applications in many branches/subbranches in neuroscience research, some caveats still remain as previously mentioned (see Figure 11).

First, and probably, the biggest limitation of OCT is its penetration depth. Usually, OCT's depth penetration is limited within 2-3 mm. Though it is better than some microscopic methods, it is less than any other existing neuroimaging methods such as MRI, fMRI, and PET. Even other optical methods such as NIRS have an imaging depth of 10–15 mm. As previously mentioned, this major disadvantage has confined OCT's potential in animal model brain imaging. Though cranial windows can be implemented in experimental cases only, noninvasive* in vivo* human brain imaging with OCT is still far-fetched.

Second, OCT has a limited field of view (FOV) which somehow slows down its clinical acceptance in angiographic imaging. However, recently Xu et al. (2016) have reported an SD-OCT system with an FOV of about 750 mm^2^ [111]. And in case of neurosurgery, Finke et al. (2012) described the combination of OCT and a robotic driven surgical microscope to enable the complete OCT scanning of a larger area in neurosurgery [96]. Besides, the lateral resolution of typical OCT systems is ~10 *μ*m with a depth of focus of ~0.15 mm [3]. It makes diameter measurements inaccurate for those vessels smaller than 20 *μ*m in angiographic imaging. This problem can be minimized by an expensive higher-resolution system with sufficient depth of focus in order to cover the entire cortical layer, and OCT setups are currently under development that can reach a resolution up to 1 *μ*m.

Third, OCT has a bulky system which occupies half of an office desk. Though OCT system is smaller than MRI/fMRI/PET, it is bulkier and less portable than other optical methods such as NIRS. As a result, OCT cannot image neural activity or functional changes in freely moving animals. In some cases, OCT creates motion-related artifacts in retinal images of children and older-aged patient groups. Though HH-OCT can mitigate this caveat, it has not widely been used in neuroscience research like other functional OCT extensions which are previously discussed. But SD-OCT or other OCT versions that have a relatively higher speed minimize the image acquisition time and create less motion-related artifacts.

Future research should, therefore, aim at combining other imaging methods with OCT in order to avoid these limitations. Recently Murphy et al. (2016) proposed a concurrent MRI/OCT imaging system to make the brain-eye correlation in glaucoma [112]. MRI/OCT multimodal neurosurgery approaches are discussed in previous sections. Moreover, recently Giardini et al. (2016) proposed a multimodal microelectrode recording/OCT system for probe insertion guidance and endoscopic analysis of spinal cord [104]. Besides, surgical microscope integrated with OCT also holds promise to synergize future neurosurgical applications and it needs no additional place in the imaging setup. Therefore, there are ample scopes of developing and designing multimodal imaging systems with OCT for both future basic and clinical neuroscience research initiatives.

## 6. Conclusion

After OCT's invention in the 1990s, OCT is playing a crucial role in several branches of neuroscience in these days. However, because of some limitations, OCT cannot fully substitute existing imaging modalities (e.g., MRI, EEG, and PET) used in neuroscience research. Nevertheless, OCT can be used as a supportive or complementary tool to existing methods. As a micrometer-scale imaging system with a high speed and low cost, OCT already gained huge popularity and has shown its applications in neuroimaging, neurosurgery, neurology, neuroembryology, neuropathology, and so on. The growth of both the research and commercial industry backing OCT will continue to yield advances in performance and broaden access to the instrumentation, further driving adoption. Future research initiatives should aim at improving OCT's depth penetration, system miniaturization, and developing multimodal systems which may yield new applications of OCT in neuroscience. Not surprisingly, it is high time that today's neuroscience community accepted OCT as a regular imaging modality like other existing modalities are.

## Figures and Tables

**Table 1:** Basic principles of some of the OCT techniques used in neuroscience research.

OCT techniques	Basic principles
Time domain OCT (TD-OCT)	A moving mirror in the reference arm, which centers the interference signal on a fixed Doppler frequency. Coherent demodulation, with a lock-in amplifier set to this frequency, enables detection of interference fringes produced by light scattered from the specimen

Fourier domain OCT (FD-OCT)	Data is acquired from the whole sample depth simultaneously with a fixed path length in the reference arm

Doppler OCT (D-OCT)	Speed of a moving particle is measured by detecting frequency shifts of the light scattered by the particle

Polarization-sensitive OCT (PS-OCT)	Sample is exposed to light from multiple polarizations to measure birefringence

Spectroscopic OCT (S-OCT)	Wavelength-dependent absorption and light scattering are used to elucidate function

See [31].

**Table 2:** Comparison of spatiotemporal resolution of OCT with other neuroimaging techniques.

Techniques	Spatial resolution	Temporal resolution	References
Magnetic resonance imaging (MRI)	Millimeter, in some cases (ultrahigh field) submillimeter	Can be used to track longitudinal changes	[1]

Functional MRI (fMRI)	Millimeter	Seconds	[1]

Magnetic resonance spectroscopy (MRS)	Centimeter	Minutes	[1]

Positron emission tomography (PET)/single positron emission and computed tomography (SPECT)	Centimeter (SPECT) to millimeter (PET)	Minutes	[1]

Magnetoencephalography (MEG)	Centimeter	Milliseconds	[1]

Electroencephalography (EEG)/event-related potentials (ERP)	Centimeter	Milliseconds	[1]

Near-infrared spectroscopy (NIRS)	Centimeter	Milliseconds	[2]

Optical coherence tomography (OCT)	Micrometer	Milliseconds	[20, 38]

**Table 3:** Disadvantages of some current neural recording techniques.

Techniques	Limitations
Microelectrode technologies	Vulnerable to environmental electrical noises and artifacts caused by electrical stimulation and neural recording and unable to reliably record chronic neural activity in most cases

EEG, MEG, thermal Imaging	Limited spatial resolution and/or temporal resolution

PET, fMRI, diffuse optical tomography (DOT)	Low temporal resolution, huge size, and expensive

**Table 4:** Current status of studying central nervous system (CNS) diseases with OCT.

Central nervous system (CNS) diseases studied with OCT	Study type (target)	Applications	References
Multiple sclerosis (MS)	Clinical (human retina)	Diagnostic biomarker development, measuring outcome of drug trials	[64, 65]

Optic neuritis (ON)	Clinical (human retina)	Diagnostic biomarker development, measuring outcome of drug trials	[17, 66, 67]

Parkinson's disease (PD)	Clinical (human retina)	Diagnostic biomarker development, measuring disease severity	[26, 68]
	Nonclinical (rodent brain)	Guiding deep brain stimulation therapy	[69]

Alzheimer's disease (AD)	Clinical (human retina)	Diagnostic biomarker development	[26, 70]

Schizophrenia	Clinical (human retina)	Diagnostic biomarker development	[25, 71]

Optic nerve edema	Clinical (human retina)	Diagnostic biomarker development	[25]

Optic neuropathies (hereditary/toxic/nutritional)	Clinical (human retina)	Diagnostic biomarker development	[25]

Glaucoma	Clinical (human retina)	Diagnostic biomarker development, measuring outcome of drug trials	[25, 72]

Amblyopia	Clinical (human retina)	Studying retinal involvement	[25]

Neurosarcoidosis	Clinical (human retina)	Studying retinal involvement	[25]

Obstructive sleep apnea-hypopnea syndrome	Clinical (human retina)	Studying retinal involvement	[25]

Friedreich's ataxia	Clinical (human retina)	Studying retinal involvement	[25]

Amyotrophic lateral sclerosis (ALS)	Clinical (human retina)	Diagnostic biomarker development	[73]

Gaucher disease	Clinical (human retina)	Diagnostic biomarker development	[74]

Bipolar disorder	Clinical (human retina, ganglion cell layer)	Diagnostic biomarker development	[75]

Sialidosis	Clinical (human retina)	Studying retinal involvement	[76]

Stroke (hemorrhagic/ischemic)	Nonclinical (rodent brain)	Studying stroke recovery	[3, 77–79]

Epilepsy	Clinical (human retina)	Studying drug resistance, studying retinal involvement	[80, 81]
	Nonclinical (rodent brain)	Epilepsy mapping	[82]

Traumatic brain injury (TBI)	Nonclinical (rodent brain)	Studying TBI recovery	[3, 83, 84]

Brain tumor	Clinical (human brain, ex vivo human brain tissue)	Intraoperative neurosurgery guidance, diagnostic assessment	[5, 18, 85]
	Nonclinical (rodent brain)	Intraoperative neurosurgery guidance	[18, 22, 86]

Usher syndrome	Clinical (human retina)	Studying retinal involvement	[87]

Huntington's disease	Clinical (human retina)	Studying retinal involvement	[88]

Wilson's disease	Clinical (human retina)	Studying retinal involvement	[89]

Migraine	Clinical (human retina)	Diagnostic biomarker development	[25]
